# Neutrophil extracellular traps offer a new therapeutic target for elephant endotheliotropic herpes virus-hemorrhagic disease (EEHV-HD)

**DOI:** 10.1038/s42003-026-10375-w

**Published:** 2026-05-30

**Authors:** Lisa M. Abegglen, Aaron Rogers, Gareth Mitchell, C. Bradley Nelson, Madison I. Sanborn, Ryan Kennington, McKenna Rogers, Virginia R. Pearson, Miranda Sharp, Lauren L. Howard, Erin Latimer, Jennifer A. Landolfi, Christine Molter, Erika Crook, Wendy Kiso, Dennis Schmitt, Paul D. Ling, Kimberly Martinod, Joshua D. Schiffman

**Affiliations:** 1 Department of Pediatrics, University of Utah, Salt Lake City, UT USA; 2 Hunstman Cancer Institute, University of Utah, Salt Lake City, UT USA; 3 Peel Therapeutics, Inc., Salt Lake City, UT USA; 4 Program in Blood Cell Development and Function, Fox Chase Cancer Center, Philadelphia, PA USA; 5 North American EEHV Advisory Group, Azle, TX USA; 6 Elephant Herpesvirus Laboratory, Smithsonian’s National Zoo and Conservation Biology Institute, Washington, DC USA; 7 Zoological Pathology Program, University of Illinois, Brookfield, IL USA; 8 Houston Zoo, Houston, TX USA; 9 Utah’s Hogle Zoo, Salt Lake City, UT USA; 10 Center for Applied Reproductive & Emerging Sciences, Fort Worth Zoo, Fort Worth, TX USA; 11 Department of Animal Science, William H. Darr College of Agriculture, Missouri State University, Springfield, MO USA; 12 Department of Molecular Virology and Microbiology, Baylor College of Medicine, Houston, TX USA; 13 Aab Cardiovascular Research Institute, Department of Medicine, University of Rochester School of Medicine and Dentistry, Rochester, NY USA

**Keywords:** Mechanisms of disease, Coagulation system

## Abstract

Elephant populations are threatened by elephant endotheliotropic herpesvirus-hemorrhagic disease (EEHV-HD), a fast-acting infection that often kills very young elephants before they reach maturity. Because it strikes calves at a critical age for survival of the species, EEHV-HD is a major challenge for global conservation. Understanding how the disease causes such severe illness is essential to improving outcomes. In other hemorrhagic diseases, dangerous blood clots form when immune cells release sticky webs of DNA called neutrophil extracellular traps (NETs). We investigate whether this process occurs in elephants with EEHV-HD. Here we show, for the first time, that elephant heterophils (neutrophils) release NETs when stimulated, including by plasma from sick elephants. Tissue samples from fatal cases show large amounts of NETs, suggesting they play a harmful role in disease progression. Encouragingly, known NET inhibitors prevent this response in vitro. Our findings reveal that uncontrolled NET formation likely contributes to EEHV-HD and identify a new potential treatment strategy. Blocking NETs could be combined with existing therapies to improve survival of affected calves, offering a promising way to support elephant health and conservation.

## Abstract (web summary)

Elephant heterophils form neutrophil extracellular traps (NETs) in response to elephant endotheliotropic herpesvirus hemorrhagic-disease plasma. NET formation can be blocked by an inhibitor called BMS-P5, suggesting a potential therapeutic strategy.

## Introduction

Elephant endotheliotropic herpesviruses (EEHV) are viruses endemic to both Asian and African elephants (*Elephas maximus* and *Loxodonta africana*) worldwide^1–9^. Seven species and multiple subtypes of EEHV are known to infect elephants, and all elephants are believed to carry some of these viruses as latent infections^2,6,10–14^. A properly primed immune system protects elephants from disease when these viruses become activated^12,15^. However, the immature immune system and loss of maternally derived antibodies in young elephants, as well as EEHV naive or compromised immune systems of adult elephants can leave them susceptible to rapidly increasing EEHV in circulation (viremia) and to illness or death from disease^6,16,17^. Exponentially increasing EEHV viremia can cause a highly fatal hemorrhagic disease (EEHV-HD) that primarily affects young, immunologically naive elephants of both species^3,5,6,9,13,14,18^. EEHV-HD occurs in elephants under human care and free ranging elephants. The endangered status of Asian and African elephants^19,20^ makes EEHV-HD-related mortality a significant conservation concern.

Clinical signs of EEHV-HD include lethargy, altered mental status, decreased food or water intake, lameness/stiffness, cranial or front limb edema, petechial hemorrhages, and cyanosis of the tongue^9,13,21^. Severe cases progress rapidly, leading to internal hemorrhaging and organ failure often within 1–7 days after clinical signs of illness are observed^18^. Not all elephants with EEHV viremia develop EEHV-HD, but for those who do, death is frequently the outcome^6,7^. Extensive efforts to treat affected elephants have increased survival. Treatments include fluids, antiviral medications, analgesics, and transfusion with plasma from EEHV seropositive elephants^5,7,14,22,23^. Because each treatment has not been tested in clinical trials, it is challenging to determine which are beneficial. The epidemiology and pathophysiology that influence EEHV viremia and progression to EEHV-HD are currently under global investigation in both free ranging and managed elephant populations^4,8,24–26^. Despite the need for more knowledge, it is clear from case review that early detection and intervention are crucial for successful treatment outcomes^7,14,23,27^. Vaccine development is underway, and effective vaccines will protect some elephants from EEHV-HD induced by rapidly increasing viremia and save lives^28–30^. Even as effective vaccines for both Asian and African elephants become available, a tremendous need exists to better understand disease progression from viremia to fatal hemorrhagic disease. Unvaccinated elephants and elephants with ineffective responses to vaccination will still require effective treatment. With a better understanding of the pathophysiology leading to severe disease and death, opportunities for therapeutic intervention may become clear.

In the context of viral infections, sepsis can occur due to the body’s overwhelming immune response leading to widespread inflammation and organ dysfunction^31^. Evidence suggests that sepsis occurs in the context of EEHV-HD^32–34^. Like sepsis observed in other species, elephants with severe cases of EEHV-HD develop hypotension, tachycardia, and altered mental status^21,35^. Consistent with this model, a recent study provided direct evidence of a pathogen-induced cytokine storm in EEHV-HD, demonstrating elevated expression of IFN-γ, IL-6, and IL-10 in blood or tissues from elephants with high viral loads^34^. Additional studies found increased expression of pro-inflammatory cytokines in EEHV-HD affected tissues^33^ and evidence of disseminated intravascular coagulopathy (DIC)^32^, an end-stage phenomenon also observed in humans with sepsis^36^. EEHVs infect capillary endothelial cells, and virus-mediated endothelial damage leads to increased vascular permeability and leakage with severe widespread edema and hemorrhage^33,37^. DIC is characterized by widespread activation of the clotting cascade, resulting in the formation of blood clots throughout the body’s small blood vessels^38^. This can lead to multiple organ damage and failure due to restricted blood flow, as well as a depletion of clotting factors and platelets that increases the risk of severe bleeding^39^. Vascular compromise is known to influence the development of sepsis in other diseases^40^. In human patients, the release of pro-inflammatory cytokines and activation of coagulation pathways further exacerbate systemic inflammation and microvascular thrombosis in DIC^41^. Early interventions used to treat EEHV-HD include treatments with efficacy against sepsis in human patients such as fluid resuscitation, vasopressor support, antibiotics to target secondary bacterial infections, and transfusion of blood products to address coagulopathy^42^.

Understanding the role of dysregulated immune responses in the pathogenesis of EEHV-HD remains crucial for implementing effective management strategies and improving survival rates in affected elephants. Discovery of underlying mechanisms that contribute to DIC and microthrombi in EEHV-HD may reveal novel targets for therapeutic interventions to mitigate EEHV-HD’s fatal impact on elephants and conservation. In humans, dysregulated neutrophil responses are known to exacerbate virus induced disease and contribute to death^43,44^. When neutrophils are activated, they can release neutrophil extracellular traps (NETs). NETs are webs of decondensed chromatin with attached enzymes to capture and degrade pathogens^45^. While NETs play a beneficial role as a first response to control infection, dysregulated NET release contributes to the pathophysiology of DIC and many diseases^45^. For example, neutrophils can be activated to form NETs in response to a variety of stimuli including pathogens, immune activation, endothelial cell damage, activated platelets, and coagulation factors leading to induction of clotting, which can further damage endothelial cells and exacerbate bleeding^46,47^. NETs are also known to directly damage vascular endothelial cells and amplify inflammation, further exacerbating the coagulopathy seen in DIC^48–50^. We previously described a role for NETs in the pathogenesis of COVID-19 in humans, including microthrombosis associated with COVID-19 induced acute respiratory distress syndrome (ARDS)^44^. EEHV-HD causes widespread hemorrhage and coagulopathy with increased microthrombosis^32,33,37^, a histologic finding suggestive of NET-mediated disease. Here, we identified increased NET formation in EEHV-HD affected tissues and demonstrated the ability of elephant heterophils (functionally equivalent to neutrophils^15^) to release NETs. Our description of NETs for the first time in elephant cells, including their presence in EEHV-HD affected tissues, expands our knowledge of the pathological mechanisms involved in this devastating disease. We introduce elephant NETs as a new therapeutic target to potentially improve outcomes in affected elephants.

## Results

### Elephant heterophils form extracellular traps in response to known NET inducing stimuli

**Fig. 1:**
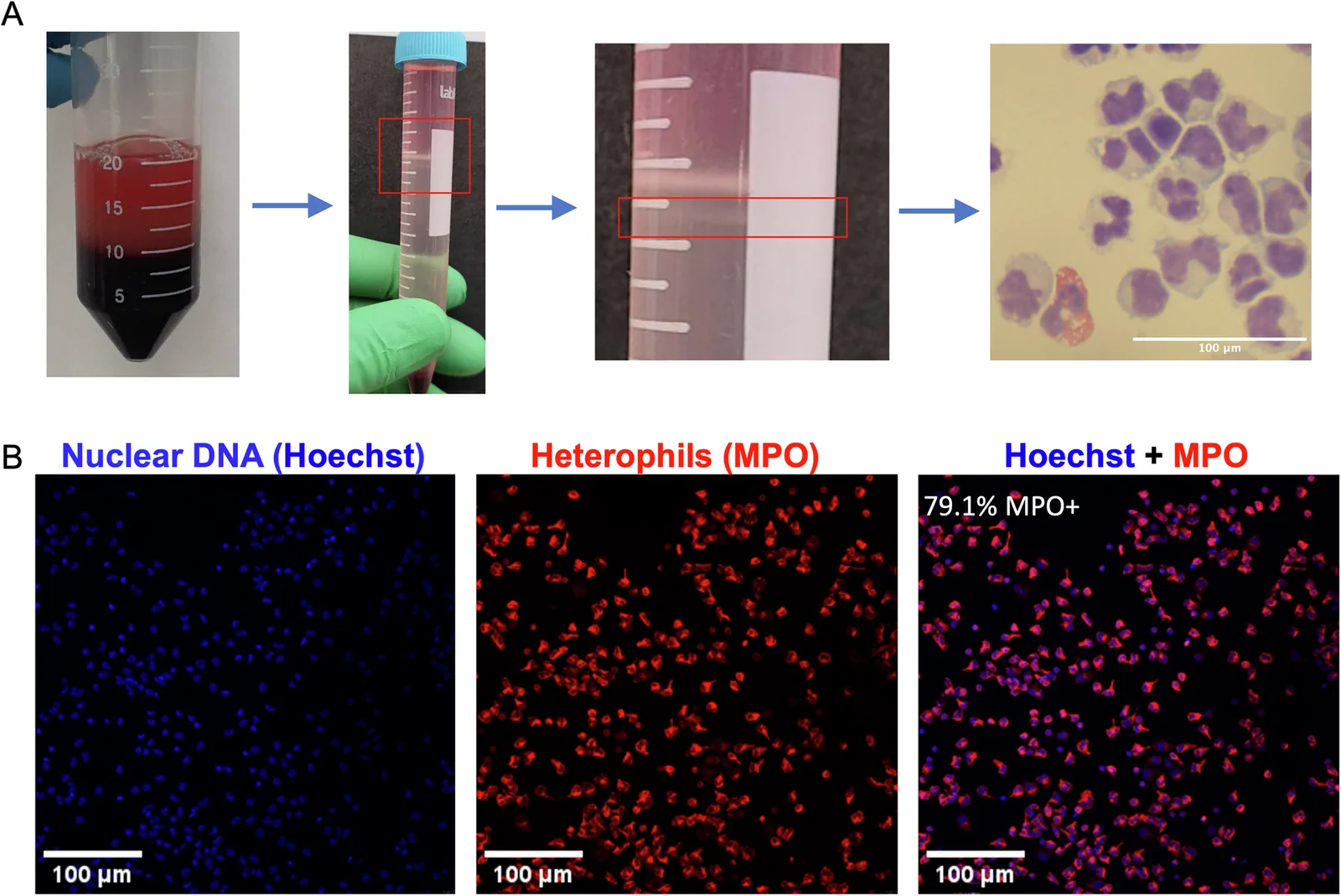
Elephant heterophil isolation. **A** Whole venous blood was collected in EDTA tubes. Following red blood cell sedimentation, white blood cells were separated by density gradient centrifugation. The bottom layer (granulocytes) of white blood cells was analyzed by microscopy-images of cytospin preparations. Heterophils were identified by nuclear structure. **B** The percent of heterophils in the isolated granulocyte layer was determined by staining for myeloperoxidase (MPO) expression. MPO+ cells/Total cells (Hoechst) was 79.1%. MPO staining appears predominantly perinuclear, consistent with localization to azurophilic granules in resting neutrophils. Granular staining is less apparent in the panel due to image resolution. High-resolution imaging demonstrated granular MPO staining in elephant heterophils (Supplementary Fig. 1).

**Fig. 2:**
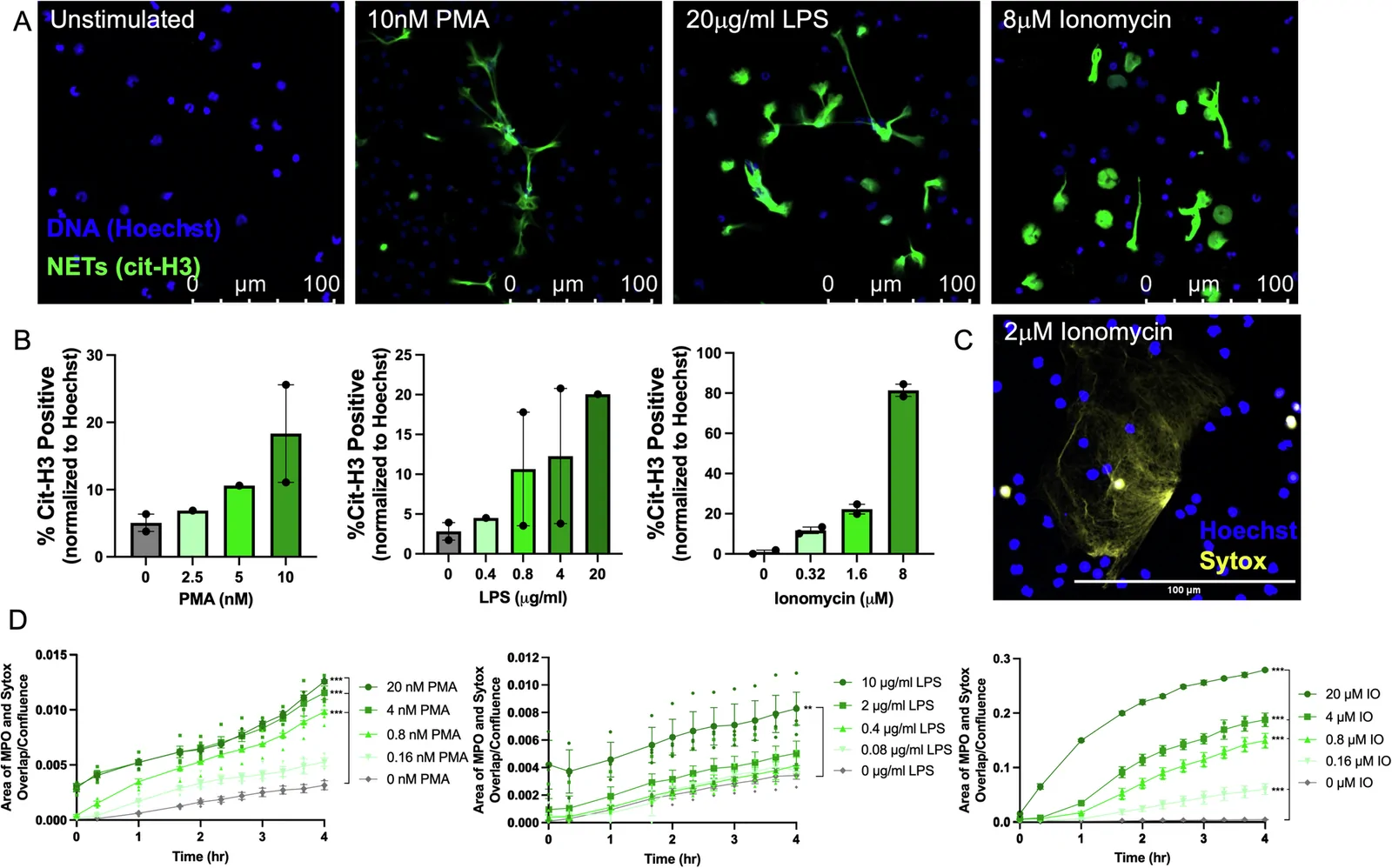
African elephant heterophils formed NETs in response to stimuli. **A** Images of African elephant heterophils unstimulated, stimulated with 10 nM PMA, 20μg/ml LPS or 8μM ionomycin. Nuclear DNA (Hoechst) is in blue and citrullinated histone H3 (citH3) is in green. **B** Dose-dependent response of African elephant heterophils (*n* = 2) to PMA, LPS, and ionomycin stimulation. Percent of citH3 cells/total cells (Hoechst) is graphed. Dots represent replicate experiments performed with blood drawn on different days from two different individuals. The representative images in (**A**) were selected to illustrate NET structure rather than response magnitude; quantitative assessment of NET formation across conditions are shown in (**B**). Data are shown descriptively and were not subjected to statistical testing due to limited sample size. **C** NET release from an African elephant heterophil stimulated with 2 μM ionomycin measured with a cell impermeable dye, Sytox Orange (yellow), to label extracellular DNA and DNA in cells with compromised membranes, and a cell permeable dye, Hoechst 33342 to label intracellular DNA (blue). **D** African elephant heterophils responded to increasing doses of stimuli over time as measured by real-time microscopy. Fluorescence time-course data were quantified as area under the curve (AUC) for each technical replicate well (*n* = 4 per condition) and analyzed by ordinary one-way ANOVA with Dunnett’s multiple comparisons test versus untreated control. PMA concentrations ≥0.32 nM showed significantly increased AUC compared to untreated (*p* < 0.001), whereas 0.16 nM showed a non-significant upward trend. LPS stimulation resulted in a significant increase in AUC at the highest concentration tested (20 µg/mL; *p* < 0.001). Ionomycin induced a significant increase in AUC, with all concentrations tested (≥ 0.16 µM) significantly greater than untreated (*p* < 0.001).

**Fig. 3:**
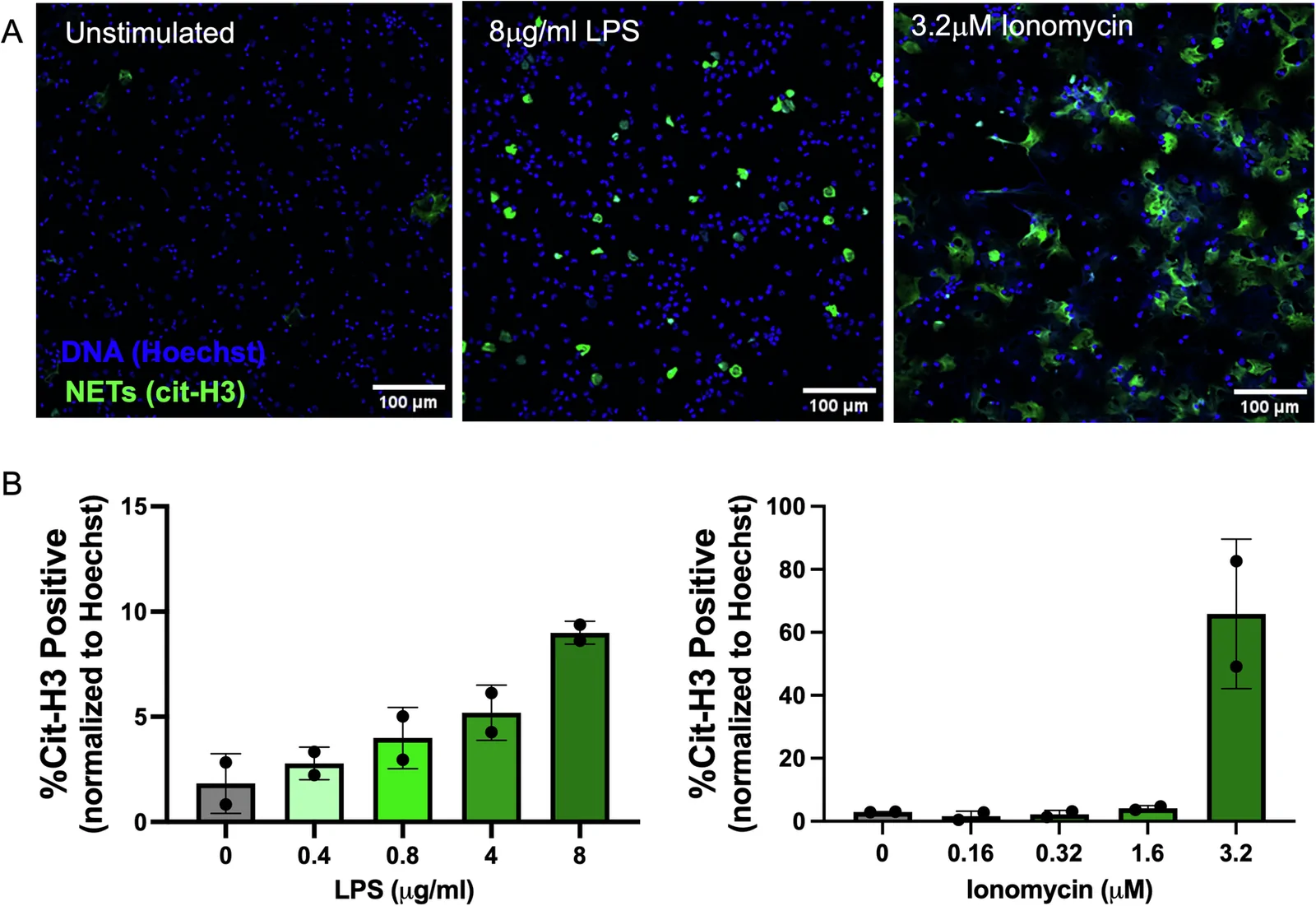
Asian elephant heterophils formed NETs in response to stimuli. **A** Images of Asian elephant heterophils (*n* = 2) that were unstimulated or stimulated with 8 μg/ml LPS or 3.2μM ionomycin. Nuclear DNA (Hoechst) is in blue and citrullinated histone H3 (citH3) is in green. **B**Dose-dependent response of Asian elephant heterophils to LPS and ionomycin stimulation. The percentage of citH3-positive cells relative to total cells (Hoechst) is shown. Dots represent replicate experiments performed with blood drawn on different days from two individual elephants. Because only two elephants were tested, data are presented descriptively.

Heterophils (functionally equivalent to neutrophils^15^) from both Asian and African elephants were enriched from fresh whole blood by red blood cell sedimentation and granulocyte isolation by density gradient centrifugation (Fig. 1A). Heterophils comprise 40% of the circulating white blood cells of elephants^51^, and we used a similar isolation approach that previously yielded heterophils with high viability (99%) and purity (98%)^52^. In the current study, heterophil viability was 97% and purity was 79% after enrichment (Fig. 1B). Processing was initiated within 30 min of blood draw due to the short half-life of neutrophils/heterophils^53^. Attempts to measure NET formation with stimulation failed if blood was not processed within 30 min of collection, due to high heterophil death and release of NETs in the untreated control samples. Enriched heterophils were treated with stimuli known to induce NET formation in other species: phorbol 12-myristate 13-acetate (PMA), and lipopolysaccharide (LPS), and ionomycin^54^. When neutrophils form NETs, chromatin decondenses and histone H3 becomes citrullinated^55^. To determine if stimulated elephant heterophils form and release NETs, immunofluorescence was performed to measure expression citrullinated histone H3 (citH3). Minimal citH3 expression was observed in unstimulated cells, and citH3 expression increased with increasing doses of stimuli in African (Fig. 2A, B) elephant heterophils. Consistent with findings in human and murine neutrophils, ionomycin induced higher citH3 signal than PMA or LPS, likely reflecting its potent calcium ionophore activity. At higher concentrations, ionomycin has also been reported to compromise membrane integrity, which may contribute to elevated citH3 signal^56,57^. As a secondary measure of NET formation and release from cells, intracellular DNA and extracellular DNA were visualized in blue and yellow, respectively, after African elephant heterophils were treated with ionomycin (Fig. 2C). Large webs of DNA (NETs) were released from heterophils. NET formation increased overtime in a dose-dependent manner when African elephant heterophils were treated with PMA, LPS, or ionomycin as measured in a third assay by real-time microscopy for overlap in DNA and myeloperoxidase (MPO), an enzyme in heterophil granules that is attached to the DNA when chromatin decondenses to form NETs (Fig. 2D). Asian elephant heterophils also increased citH3 expression with increasing doses of LPS and ionomycin (Fig. 3A, B). Elephant heterophils formed more NETs in response to stimulation compared to unstimulated heterophils. Given the limitations related to collecting and processing fresh samples and the lack of access to real-time live-cell imaging, detecting citH3 by confocal microscopy was the most practical method for visualizing and quantifying NETs, as it allowed for cell fixation after stimulation.

### Plasma from EEHV-HD affected elephants stimulates NET formation and release

**Fig. 4:**
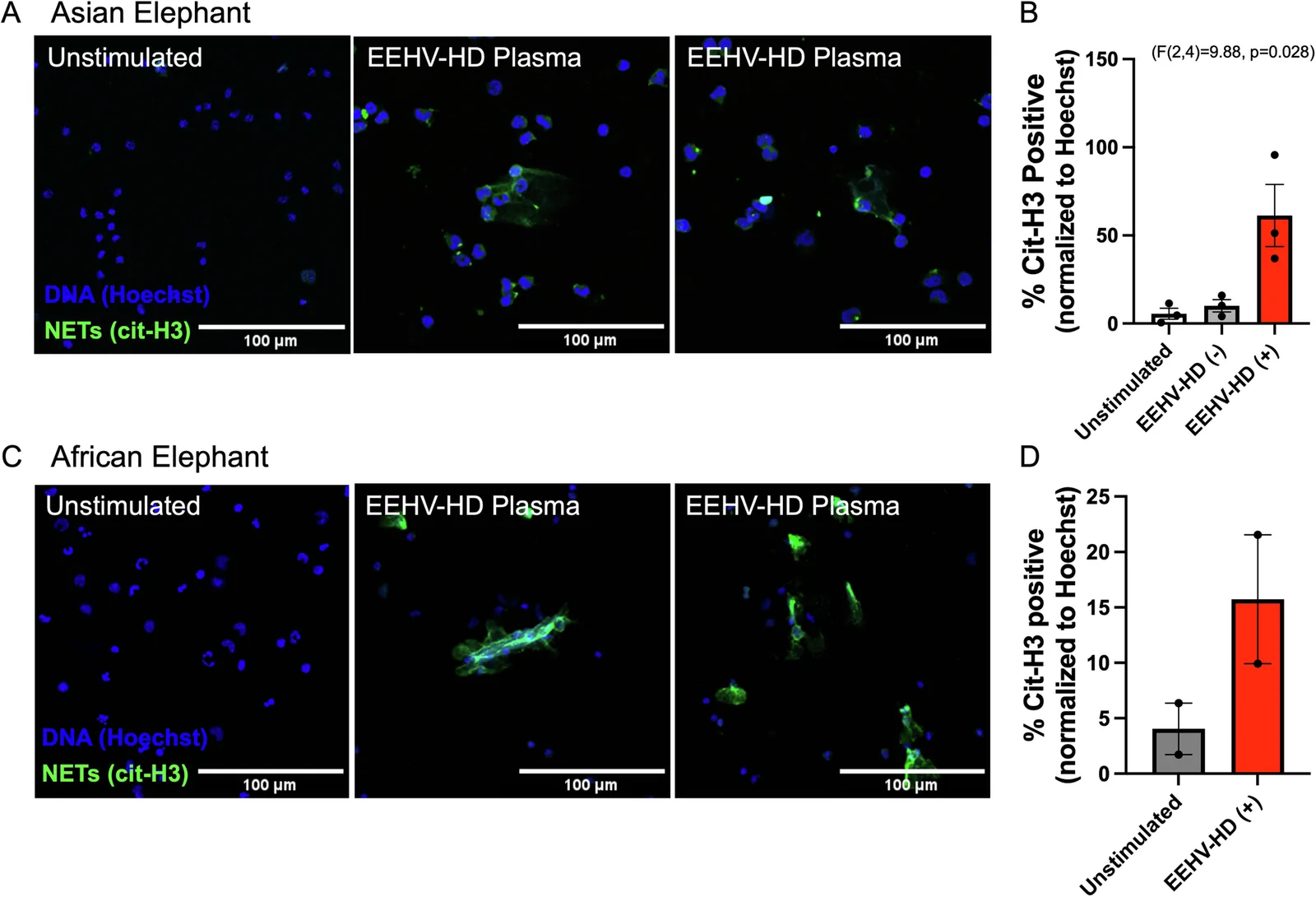
Asian and African elephant heterophils formed NETs in response to plasma collected during active EEHV-HD. **A** Images (2 example images) and **B** quantification of Asian elephant heterophils (*n* = 3) treated with healthy or EEHV-HD positive plasma. Dots represent replicate experiments performed with blood drawn on different days from different individuals. Data were analyzed using repeated-measures one-way ANOVA (F(2,4) = 9.88, *p* = 0.028). **C** Images (2 example images) and **D** quantification of African elephant heterophils (*n* = 2) untreated or treated with EEHV-HD positive plasma. Nuclear DNA (Hoechst) is in blue and citrullinated histone H3 (citH3) is in green. The percentage of citH3-positive cells relative to total cells (Hoechst) is graphed. Dots represent replicate experiments performed with blood drawn on different days from different individuals. Data are shown to illustrate directional effects; statistical testing was not performed due to limited sample size.

NETs are formed and released from human neutrophils during viral infection^44,50^. To determine if factors present in the plasma during active EEHV-HD stimulate elephant heterophils to form NETs, plasma was collected from a very sick Asian elephant during active EEHV-1A viremia (confirmed by PCR) with symptoms of EEHV-HD who ultimately died from hemorrhagic disease, determined by the presence of lesions indicative of HD detected histologically in tissues collected at necropsy. When treated with this EEHV-HD positive plasma, healthy Asian elephant heterophils (*n* = 3) formed and released NETs, while control plasma collected from an Asian elephant negative for EEHV viremia did not stimulate NET formation (F(2,4) = 9.88, *p* = 0.028) (Fig. 4A, B). Next, we treated African elephant heterophils with the same EEHV-HD positive plasma. In African elephant heterophils (*n* = 2), EEHV-HD plasma increased NET formation relative to unstimulated conditions in both animals (Fig. 4C, D). Due to limited samples size, these data are presented descriptively and were not subjected to formal statistical testing.

### NETs are present in EEHV-HD affected tissues

**Fig. 5:**
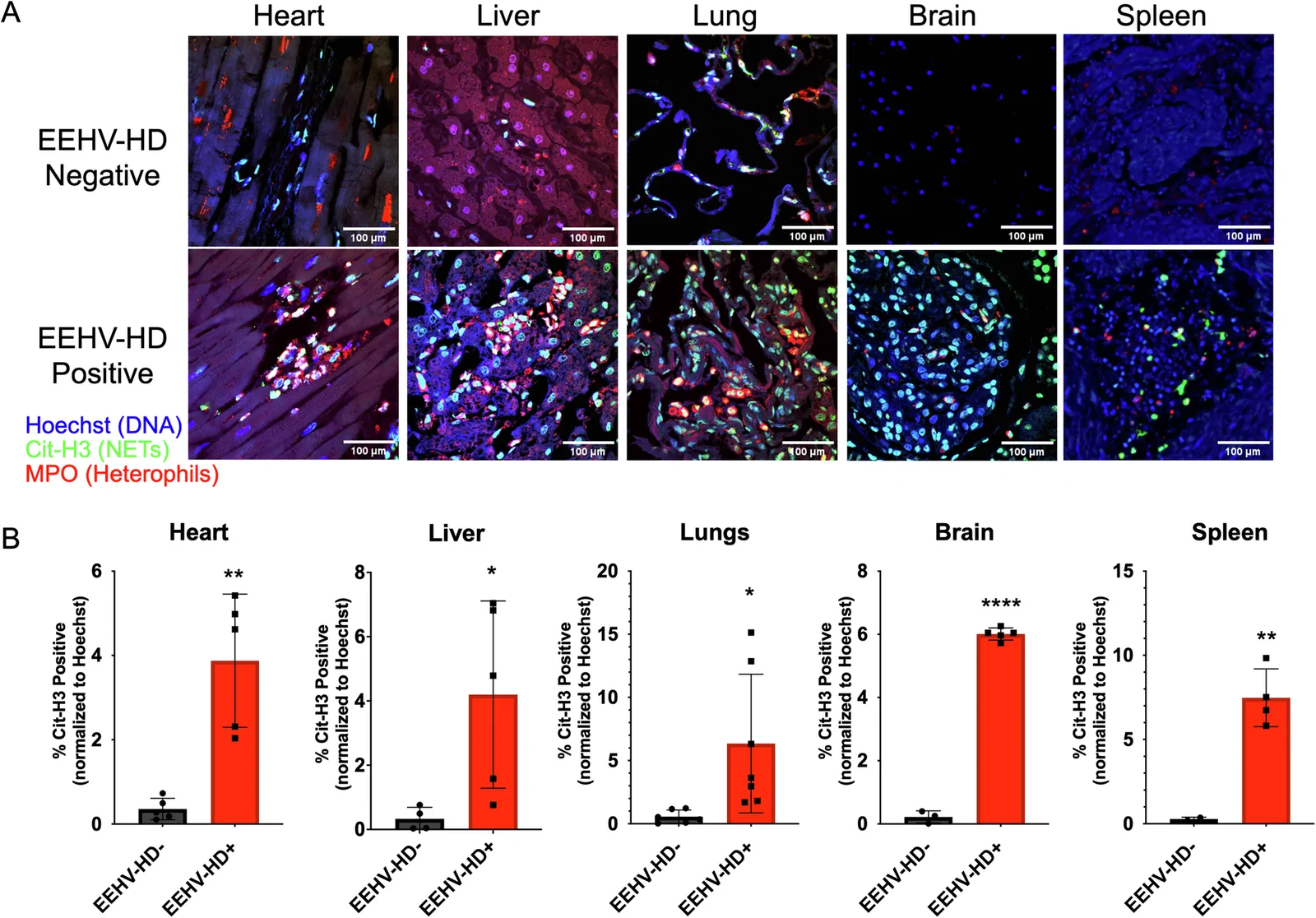
NETs formed in tissues from EEHV-HD affected Asian elephants. **A** Representative images of necropsy tissues from Asian elephants that died of EEHV-HD and control elephants that died of other causes. Nuclear DNA is shown in blue (Hoechst), heterophils are shown in red (MPO), and NETs are shown in green (citH3). **B** Quantification of NET formation in tissues. Each dot represents tissue from individual Asian elephants listed in Table 1 (EEHV-HD- heart *n* = 5, EEHV-HD+ heart *n* = 5, EEHV-HD- liver *n* = 4, EEHV-HD+ liver *n* = 5, EEHV-HD- lung *n* = 6, EEHV-HD+ lung *n* = 7, EEHV-HD- brain *n* = 3, EEHV-HD+ brain *n* = 5, EEHV-HD- spleen *n* = 2, EEHV-HD+ spleen *n* = 4). Statistical comparisons between EEHV-HD- and EEHV-HD+ tissues were performed using an unpaired two-tailed t-test with Welch’s correction. *p < 0.05, **p < 0.005, ****p < 0.0001.

To determine if NETs could be present in tissues from elephants who succumbed to EEHV-HD, immunofluorescence to detect citrullinated histone H3, MPO, and DNA was performed on tissues collected at necropsy from elephants whose cause of death was EEHV-HD and elephants who died of other causes. Table 1 lists the species, sex, age, virus type, and tissue type of the samples tested. Tissue samples from hearts, livers, lungs, spleens, and brains were imaged for the presence of heterophils (MPO), NETs (citH3), and DNA (Hoechst) (Fig. 5A). Quantification of likely NET-forming cells and released NETs in these tissues revealed a significant increase in citH3 expression in EEHV-HD affected tissues compared to non-EEHV-HD tissues (heart *p* < 0.005, liver *p* < 0.05, lung *p* < 0.05, brain *p* < 0.0001, spleen *p* < 0.005) (Fig. 5B).

**Table 1:** Asian elephant tissues analyzed and quantified for NET formation

Species	Common name	Age (years)	Sex	Cause of death	EEHV type	Heart	Liver	Lung	Brain	Spleen
*Elephas maximus*	Asian elephant	2	M	EEHV-HD	EEHV1A	+	+	+	+	
*Elephas maximus*	Asian elephant	3	M	EEHV-HD	EEHV1A	+	+	+	+	+
*Elephas maximus*	Asian elephant	4	M	EEHV-HD	EEHV1A	+				+
*Elephas maximus*	Asian elephant	5	M	EEHV-HD	EEHV1A	+	+	+	+	
*Elephas maximus*	Asian elephant	6	F	EEHV-HD	EEHV1A				+	
*Elephas maximus*	Asian elephant	6	F	EEHV-HD	EEHV1A			+		
*Elephas maximus*	Asian elephant	9	F	EEHV-HD	EEHV1A		+	+		+
*Elephas maximus*	Asian elephant	5	F	EEHV-HD	EEHV1A	+		+		
*Elephas maximus*	Asian elephant	13	M	EEHV-HD	EEHV1A		+	+	+	+
*Elephas maximus*	Asian elephant	1	F	Other	NT	+				
*Elephas maximus*	Asian elephant	44	F	Other	NT	+				
*Elephas maximus*	Asian elephant	44	M	Other	NT			+		
*Elephas maximus*	Asian elephant	46	F	Other	NT			+		
*Elephas maximus*	Asian elephant	47	F	Other	NT		+	+		
*Elephas maximus*	Asian elephant	53	F	Other	NT	+	+	+	+	+
*Elephas maximus*	Asian elephant	60	F	Other	NT	+	+		+	+
*Elephas maximus*	Asian elephant	73	F	Other	NT	+	+		+	
*Elephas maximus*	Asian elephant	Unknown	F	Other	NT			+		
*Elephas maximus*	Asian elephant	Unknown	F	Other	NT			+		

### NET inhibitors block elephant NET formation in response to stimuli, including EEHV-HD positive plasma

**Fig. 6:**
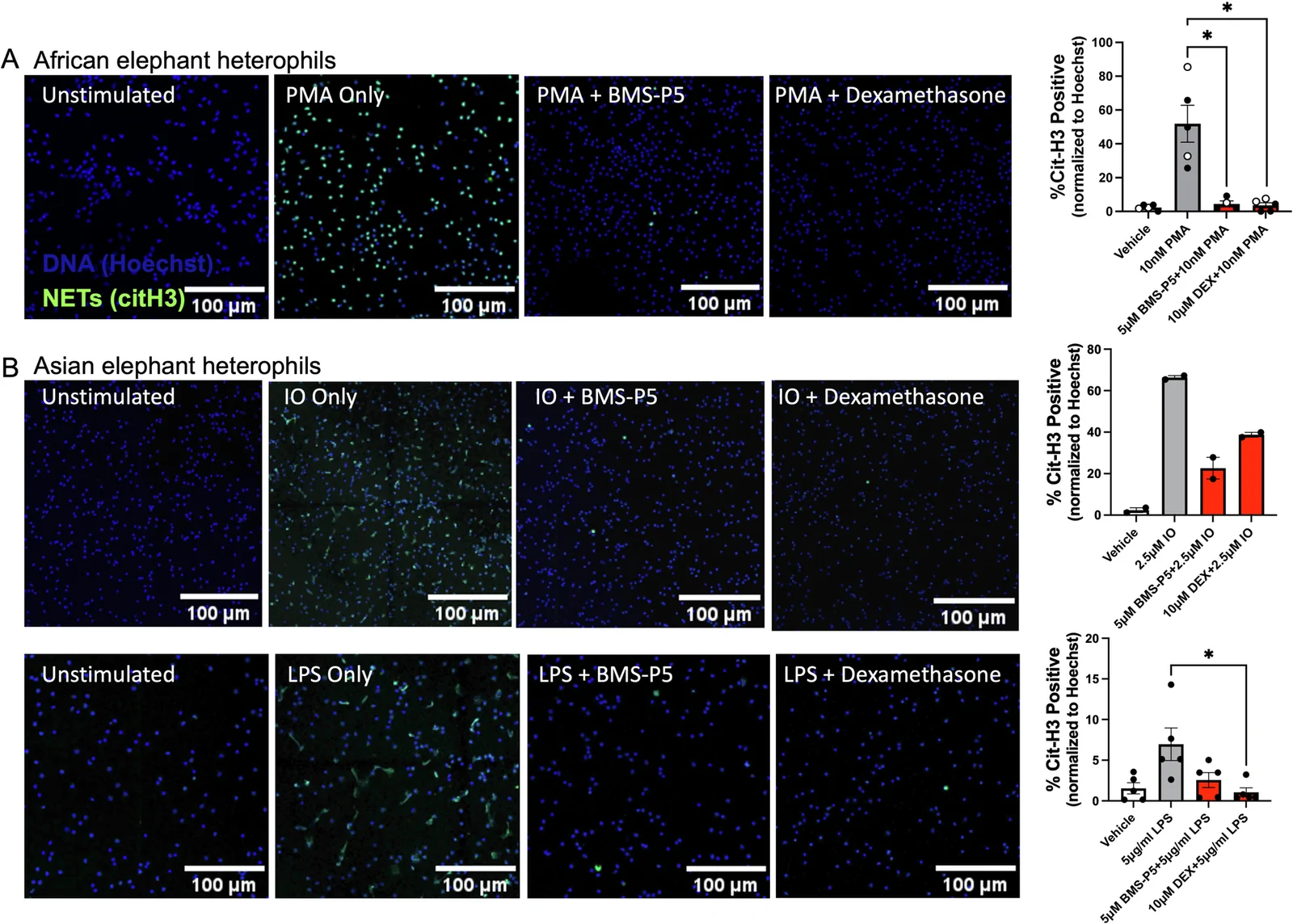
PMA, ionomycin, and LPS stimulated NET release from elephant heterophils were inhibited by BMS-P5 and dexamethasone. **A** Representative images and quantification of African elephant heterophils (*n* = 2) treated with PMA after pretreatment with either vehicle, BMS-P5, or dexamethasone. Dots represent independent biological replicates derived from two individual elephants, each sampled on multiple days (total replicates = 5). Data points corresponding to each elephant are distinguished by shaded and unshaded symbols. **B** Representative images and quantification of Asian elephant heterophils treated with ionomycin (IO) (*n* = 2) or LPS (*n* = 5) after pretreatment with either vehicle, BMS-P5, or dexamethasone. Sample sizes differed across stimulation conditions because Asian elephant heterophils could only be collected during a single on-site visit, and limited cell numbers from each animal, combined with the requirement to perform assays within 30 min of blood draw, prevented testing all conditions on every elephant. NETs were quantified and graphed. Dots on graphs represent different individual elephants tested. Planned paired comparisons were performed between stimulant + inhibitor conditions versus stimulant alone within the same elephants using paired two-tailed t-tests with Holm–Šídák correction for PMA and LPS. *p < 0.05. Statistical testing was not performed on IO stimulated cells due to limited sample size. Data are shown to illustrate directional effects.

**Fig. 7:**
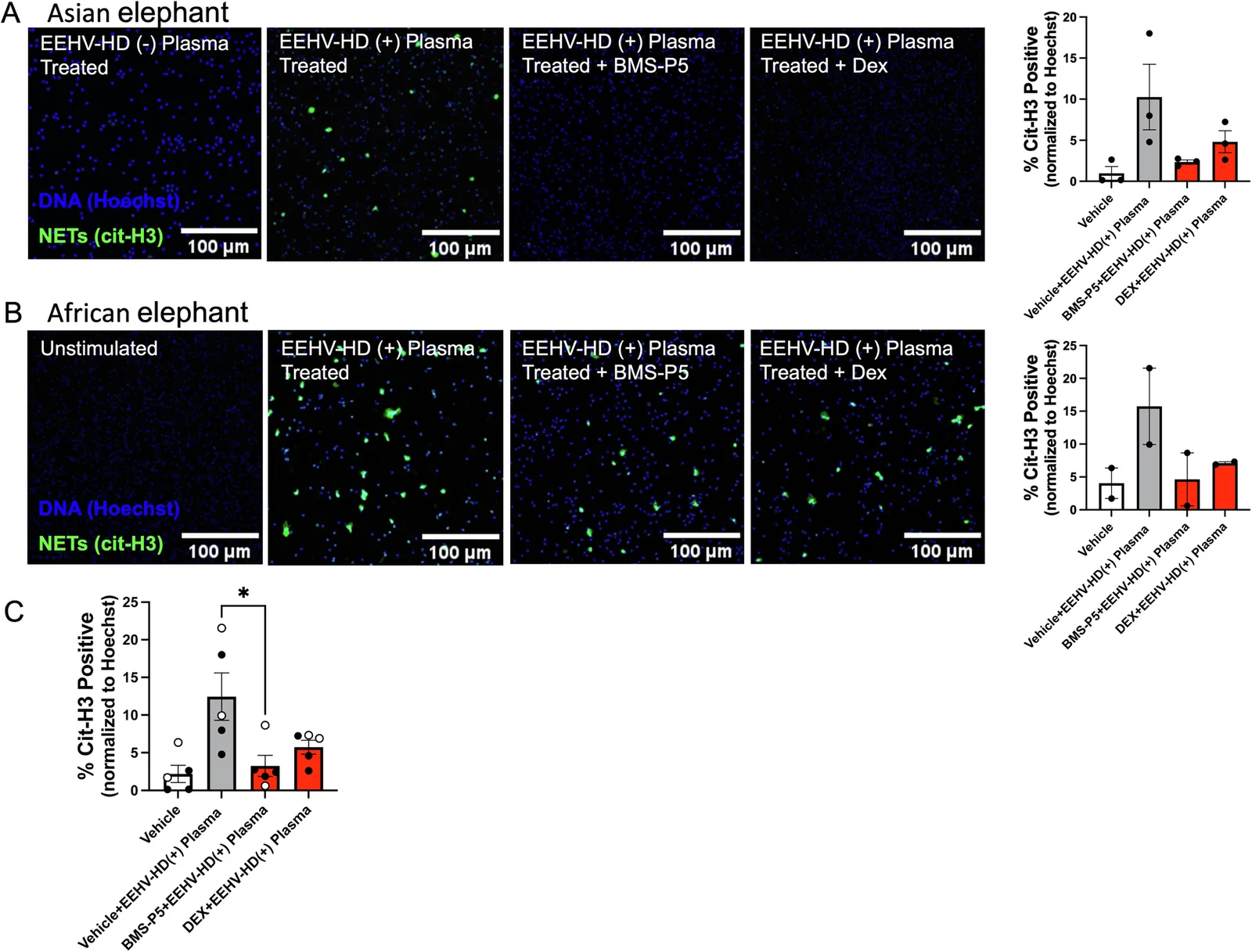
EEHV-HD positive plasma stimulated NET release from elephant heterophils was inhibited by BMS-P5 and dexamethasone. **A** Images and quantification of Asian elephant heterophils (*n* = 3) treated with plasma from an EEHV-HD affected elephant after pretreatment with either vehicle, 5 μM BMS-P5, or 10 μM dexamethasone. The control in the first box is heterophils treated with EEHV-HD negative plasma. **B** Images and quantification of African elephant heterophils (*n* = 2) treated with plasma from an EEHV-HD affected elephant after pretreatment with either vehicle, BMS-P5, or dexamethasone. The control in the first box is unstimulated heterophils. NETs were quantified and graphed. Dots on graphs represent different individual elephants tested. **C** Combined quantification of NET release and inhibition for all elephants tested. Because both species showed qualitatively similar directional responses (NET induction by EEHV-HD positive plasma and partial inhibition by BMS-P5 and dexamethasone) the datasets were pooled to provide an overall summary of inhibitor effects across all animals. Data points corresponding to Asian and African elephants are distinguished by shaded and unshaded symbols, respectively. Species-specific data are shown in (**A**) and (**B**) for transparency. Pooling was used to increase statistical power give the limited number of elephants available for testing. *p < 0.05.

In multiple lethal mouse models of sepsis and viral disease, blocking NET formation or degradation of NETs improved survival^58–61^. Multiple NET-specific inhibitors are in preclinical and clinical development for human diseases^62^. BMS-P5 is a compound under clinical development that effectively inhibits NET formation by selectively targeting PAD4, which is an enzyme critical for NET formation^63^. Another strategy to broadly suppress NET formation is through general immune suppression with steroid drugs like dexamethasone. Dexamethasone inhibits NET formation^64^ and has been shown to improve outcomes in some human viral diseases, including COVID-19^65^. To determine if BMS-P5 or dexamethasone could inhibit elephant NET formation, African and Asian elephant heterophils were treated with these inhibitors prior to stimulating with PMA, ionomycin (IO), or LPS. NET formation was measured by staining for citH3 and DNA. Decreased NET formation was observed in PMA stimulated African elephant heterophils that were pretreated with either BMS-P5 or dexamethasone (Fig. 6A) compared to those pretreated with vehicle (PMA compared to BMS-P5 + PMA *p* < 0.05, PMA compared to dexamethasone + PMA *p* < 0.05). In two Asian elephants tested with ionomycin, both BMS-P5 and dexamethasone reduced NET formation relative to ionomycin alone in a directionally consistent manner (Fig. 6B). Due to the limited sample size (*n* = 2), these data are presented descriptively and were not subjected to formal statistical testing. Decreased NET formation was also observed in LPS stimulated Asian elephant heterophils that were pretreated with either BMS-P5 or dexamethasone (Fig. 6B) compared to those pretreated with vehicle (LPS compared to dexamethasone + LPS *p* < 0.05). Next, we measured the ability of these drugs to inhibit NET formation in heterophils stimulated with EEHV-HD positive plasma. Although inhibition of NET formation appeared visually more pronounced in Asian elephant heterophils than in African elephant heterophils, both species demonstrated the same directional response, characterized by robust NET induction following EEHV-HD-positive plasma stimulation and reduced NET formation after pretreatment with BMS-P5 or dexamethasone (Fig. 7A, B). Because species-specific sample sizes were limited (Asian *n* = 3 and African *n* = 2), species-level analyses were underpowered for statistical inference. We therefore pooled data across all elephants to evaluate the overall effect of NET inhibition while retaining species-stratified plots to confirm the absence of opposing responses. In the combined analysis (Fig. 7C), pretreatment with BMS-P5 significantly reduced EEHV-HD plasma-induced NET formation (*p* < 0.05), whereas dexamethasone showed a trend toward inhibition. Together, these findings suggest that NET inhibitors can reduce EEHV-HD plasma induced NET formation across elephants.

## Discussion

We report for the first time that heterophils isolated from the blood of Asian and African elephants form NETs in response to stimulation. Significant citH3 expression, likely indicating NET formation, was also observed in tissues from elephants that succumbed to EEHV-HD, along with evidence of NETs within lesions that are likely microthrombi. These findings align with previous reports of disseminated intravascular coagulation (DIC) and microthrombosis in some EEHV-HD-affected tissues^32,33^. Given the established role of NETs in the pathogenesis of viral diseases in other species^50^, including virus-induced clotting^44^, our results suggest that NETs likely contribute to the development and lethality of hemorrhagic disease caused by EEHV viremia. We report that NET formation can be inhibited in elephant immune cells in vitro, suggesting that NETs may represent a future therapeutic target for this lethal disease that threatens the survival of elephant species. Importantly, our findings are based on in vitro and ex vivo experiments and do not directly demonstrate treatment efficacy in elephants. NET inhibition as a potential strategy to mitigate EEHV-HD will require further investigation in living elephants before being recommended for treatment.

NETs play a damaging role in inflammation-related diseases, and several therapeutic companies are actively developing approaches to inhibit their formation^66^. Although NET-specific inhibitors are not yet FDA-approved, other existing therapeutics can impact NET formation either by nonspecifically suppressing the immune response or by degrading NETs post-formation. For example, dexamethasone has shown survival benefits in humans with severe COVID-19 potentially through NET inhibition^67^. Additionally, in various models of sepsis, NET inhibition or degradation has been associated with improved survival^58–61^. Therefore, one of our study aims was to evaluate whether NET release from elephant heterophils could be inhibited, as this could inform future exploratory therapeutic strategies for elephants with EEHV-HD. We found that a compound designed to inhibit PAD4 activity (BMS-P5) and a general immune suppressor (dexamethasone) were both effective at inhibiting NET release from Asian and African elephant heterophils in vitro, including heterophils stimulated with plasma from EEHV-HD-affected elephants.

**Fig. 8:**
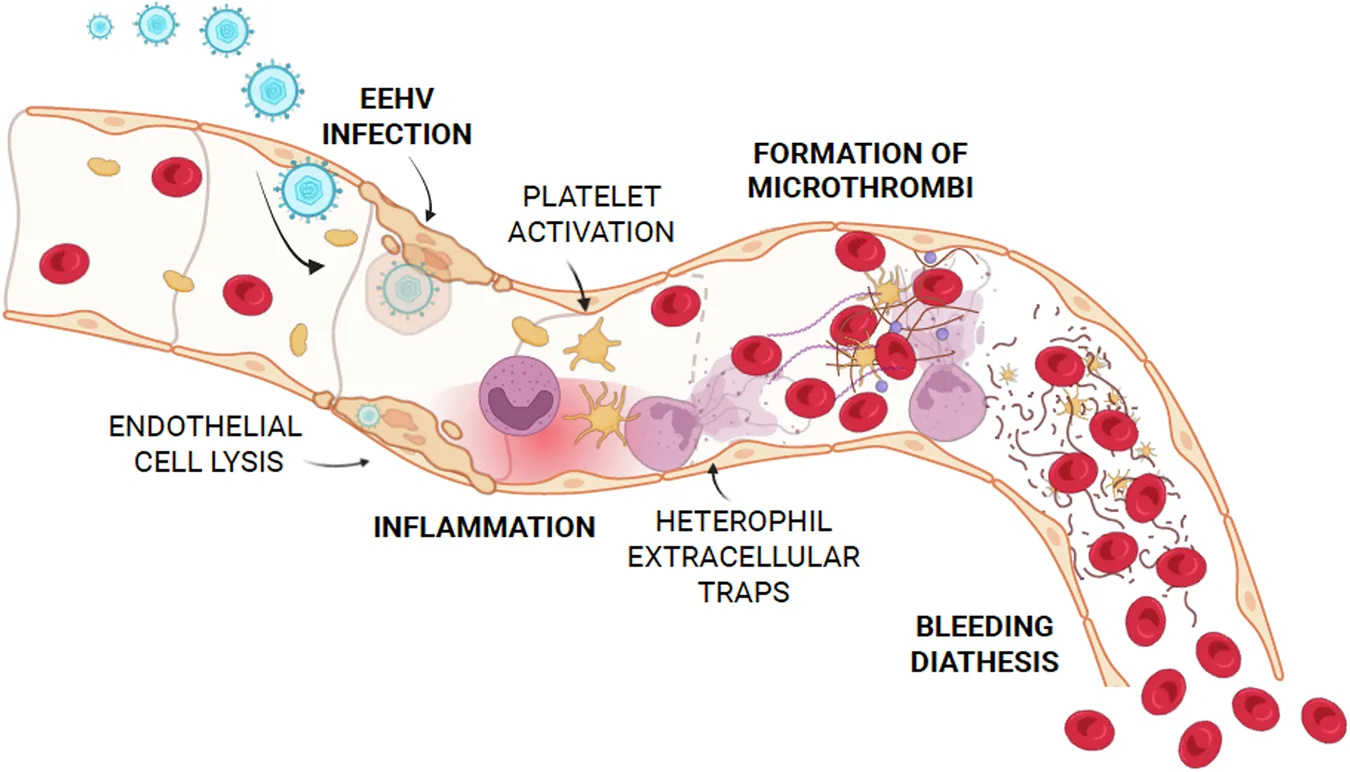
Proposed model of EEHV-HD induced NET release and downstream immunothrombosis. Schematic illustrating our working model in which EEHV-HD triggers excessive NET release, contributing the endothelial injury, inflammation, and microvascular thrombosis. This model is hypothesis-generating and based on known pro-coagulant effects of NETs in humans, together with the documented presence of microthrombi in EEHV-HD tissues. Direct evidence that NETs promote coagulation in elephants is not yet available and will require future investigation. Created in BioRender. Martinod, K. (2026).

Based on these findings and the role of NETs in virus-induced thrombosis in humans^44^, we propose a hypothesis-generating model whereby EEHVs replicate in endothelial cells lining small blood vessels leading to cell lysis. This damage activates platelets and clotting factors, triggering an inflammatory response and subsequent NET release from heterophils. The NETs then exacerbate endothelial damage and contribute to microthrombi by interacting with red blood cells, fibrin, and platelets. The formation of microthrombi and further endothelial damage likely exacerbates bleeding caused by viral lysis of endothelial cells (Fig. 8). This robust immune response, which results in fatalities more frequently in Asian than African elephants with EEHV-HD, aligns with our earlier genomic studies that demonstrated selection of TNF-alpha response pathways in the evolution of Asian more than African elephants^68^.

A direct comparison of Asian and African elephant NET formation could reveal differences related to EEHV-HD susceptibility and outcomes. Although representative images in our study may suggest morphological differences in NETs formed by Asian and African elephant heterophils, these observations cannot be interpreted biologically. The two datasets were generated at different institutions, on different days, using independent blood samples that required immediate on-site processing. NET morphology is highly sensitive to technical variables, including time from blood draw, cell handling, fixation, staining, and imaging parameters, which were necessarily distinct between the spatially separated experiments. A controlled comparison would require simultaneous access to both African and Asian elephants near a laboratory with specialized equipment and age-matched individuals, which was not feasible in this study. Future comparative studies will be valuable, and our findings highlight the need to directly assess potential species-specific differences in NET morphology and function under standardized conditions.

Our study results support NET inhibition as a new therapeutic target for EEHV-HD to decrease inflammation and impact clot formation, which potentially could improve outcomes of sick elephants. Inhibiting NET formation or degrading existing NETs has been shown to improve survival in animal models of DIC and sepsis^58–61^. Steroid drugs like dexamethasone can inhibit NET formation by suppressing inflammatory transcription factors^69^. In our study, dexamethasone effectively inhibited EEHV-HD-positive plasma induced NET formation in isolated elephant heterophils. While this finding suggests that dexamethasone may modulate NET-associated pathology, its efficacy in vivo in the context of EEHV-HD remains unproven. The broader immunosuppressive effects of dexamethasone should be carefully considered in any future clinical trial, as it may also suppress other beneficial immune responses. Case reports have described the use of dexamethasone alongside other supportive therapies in EEHV-HD-affected elephants, often as part of a comprehensive treatment plan including fluid therapy, antiviral medications, antibiotics, and supportive care^22,70^. In humans, effective dosage and duration of dexamethasone treatment vary based on disease severity and response to treatment. This variability may also be the case in elephants. In some EEHV-HD cases, dexamethasone administration was associated with clinical improvement such as fever resolution, edema reduction, and stabilization of vital signs^71^. However, treatment outcomes remain variable in elephants suffering from EEHV-HD and not all elephants will respond favorably to treatment, regardless of dexamethasone use.

While dexamethasone may alleviate inflammation, its use in EEHV-HD remains controversial among veterinarians due to concerns about potential adverse effects and its potential impact on viral replication. For instance, dexamethasone has been shown to increase the replication of cytomegalovirus, a herpesvirus that infects humans^72^. Whether dexamethasone affects EEHV replication remains unknown, partly due to the challenges of culturing EEHV in vitro^73^. The potential impact of dexamethasone on EEHV replication could be discovered by measuring viral titers before and after treatment in sick elephants. Ultimately, collecting and documenting clinical and laboratory data from elephants treated with and without dexamethasone will help clarify whether this drug offers a survival benefit in the context of EEHV-HD along with the risks of EEHV replication. In human viral infections, the timing and dosage of dexamethasone administration are critical to optimizing outcomes^74^, suggesting that similar considerations will be necessary for elephants with EEHV-HD. Further research is required to determine the optimal dose, timing, and route of administration of dexamethasone to effectively decrease NET formation, vascular damage, and immunothrombosis without exacerbating other pathogenic aspects of the disease.

In addition to dexamethasone, we found that a commercially available PAD4 inhibitor (BMS-P5) blocks EEHV-HD plasma induced NET formation. While this drug is not FDA approved and its future clinical development remains unclear, new therapeutic approaches to inhibit NET formation without affecting other immune functions are in active development for treating various inflammatory diseases, including DIC-induced thrombosis. Studies in mice demonstrated that blocking NET formation significantly improved survival in models of hemorrhagic disease, sepsis, stroke, and other conditions with symptoms similar to EEHV-HD^58–60,75^. Once NET-specific inhibitors become clinically available, targeted evaluation in EEHV-HD may be warranted. Controlled studies will be needed to determine whether inhibiting NETs improves clinical outcomes in elephants.

An FDA-approved approach that potentially mitigates the harmful effects of NETs is recombinant DNase I (dornase alpha or Pulmozyme), which digests NETs after their release. Pulmozyme is primarily used to treat cystic fibrosis and is administered via inhalation^76,77^. During the COVID-19 pandemic, aerosolized DNase I was used with partial success to block fatalities from NET-associated acute respiratory distress syndrome^44,78,79^. While lung administration may not benefit other organs affected by EEHV-HD, DNase has been explored in animal models using alternative delivery methods^80^ and is in clinical trials for sepsis for intravenous administration (IdealSEPSIS1, NCT05453695). In addition, rectally administered DNase has shown a significant survival benefit in a mouse model of sepsis^58^. This novel adaptation could be an approach worth investigating in EEHV-HD. Another potential therapeutic to consider testing in elephants is disulfiram, a drug that blocks acetaldehyde (ALDH) which may also disrupt NETs^81^.

A limitation of this study is that non-EEHV control tissues were obtained from older elephants. Age-matched controls for EEHV-HD tissues were not available because most younger elephant samples come from juvenile elephants that have succumbed to EEHV-HD making uninfected control tissue difficult to obtain for young elephants. Although age-dependent NET biology has not been studied in elephants, studies in humans and mice report increased NET formation with age, suggesting that older age is unlikely to underestimate NET levels in control tissues^82,83^. Thus, while age remains a constraint of sample availability, available evidence suggests that age-matched controls, if obtainable, would be expected to exhibit equal or lower levels of NET formation than the older control tissues analyzed in our study. Therefore, we believe the tissue used from older elephants in our study would be unlikely to diminish the observed differences between EEHV-HD and non-EEHV-HD samples. Access to fresh elephant blood samples was another technical limitation of this study. Future experiments on NETs in elephants should extend these findings by testing plasma from additional EEHV-HD-positive elephants across a larger number of heterophil donors to determine how variability in viral load, disease stage, and host inflammatory milieu influences NET induction and inhibitor responsiveness.

In conclusion, we offer the first demonstration of NET release in elephant heterophils and their potential contribution to EEHV-HD, a deadly coagulopathy in elephants. A thorough understanding of the interplay between viral infection and immune response is essential to improve outcomes for elephants affected by EEHV-HD. Ongoing efforts to characterize disease progression, facilitated by the North American EEHV Advisory Group^21^, underscore the importance of collaboration among researchers, veterinarians, elephant care experts, and pathologists worldwide. Through continued research, cooperation, and careful analysis, these research collaborations can uncover effective strategies to mitigate this devastating disease, ultimately helping to ensure the survival of elephant species.

## Methods

### Neutrophil isolation

Whole blood was collected from Asian elephants (*Elephas maximus*) and African elephants (*Loxodonta africana*) housed at Houston Zoo and Utah’s Hogle Zoo (respectively) by venipuncture into EDTA-coated vacutainer tubes under approval from Houston Zoo’s Investigative Review Committee and Utah’s Hogle Zoo Science Review Committee, respectively. We have complied with all relevant ethical regulations for animal use. An overview with representative images of heterophil isolation is shown in Fig. 1. Blood was processed to isolate elephant heterophils within 30 min of collection. Tubes were placed in an incubator for 15–30 min to sediment red blood cells (RBCs). Overlying suspension containing white blood cells (WBCs) was collected and transferred to a fresh tube. PBS was added and the tube was centrifuged at 525 × *g* for 5 min with no break to pellet the cells. The cells were resuspended in media (RPMI with 0.05% FBS heat inactivated at 70 °C for 30 min) and layered onto Mono-Poly Resolving Medium (MP Biomedicals) followed by centrifuging for 30 min at 300 × *g* in a swinging bucket rotor at room temperature with the brake off to allow proper separation. The layer of cells containing heterophils was harvested using a pipette, avoiding contamination from the other layers. Cells were stained with goat anti-human/mouse MPO (1:67, AF3667; R&D Systems) and DyLight 649-conjugated anti-goat secondary antibodies. ProLong Glass Antifade Mountant formulated with NucBlu (Hoechst 33342) was used to stain DNA (P36981; Invitrogen). MPO+ cells/total cells (Hoechst) was calculated to determine the percent of heterophils in the isolated granulocyte layer. Although MPO staining may appear diffuse at lower magnification, high-resolution imaging demonstrates granular cytoplasmic MPO localization in elephant heterophils (Supplementary Fig. 1).

### In vitro neutrophil/heterophil extracellular trap formation assays

For confocal imaging, 8-well glass chamber slides were coated with poly-L-lysine. Half a million cells were seeded in each well in RPMI media with 0.05% heat inactivated FBS. FBS was inactivated at 70 °C for 30 min to inactivate DNase in serum. Cells were stimulated with the indicated concentrations of PMA (phorbol 12-myristate 13-acetate), LPS (lipopolysaccharide), ionomycin, or EEHV-HD positive plasma (from an Asian elephant that succumbed to EEHV-1A) for 2 h at 37 °C. The cells were then fixed and stained for confocal imaging. For inhibition experiments, cells were pretreated with the concentrations shown in the respective figures of inhibitors (dexamethasone from Selleck Chemicals S1322 or BMS-P5 from Cayman Chemical 33581) for 30 min prior to adding stimulants at the indicated concentrations. Cells were stained with rabbit anti-human citrullinated histone H3 (1:400, ab5103; Abcam) with AlexaFluor488-conjugated anti-rabbit secondary antibodies. ProLong Glass Antifade Mountant formulated with NucBlu (Hoechst 33342) was used to stain DNA (P36981; Invitrogen). Images were captured by confocal microscopy (Leica SP8) and analyzed using FIJI/Image-J (version 2.1.0/1.53c; NIH). Because Asian elephant heterophils must be tested within 30 min of blood collection and assays could only be performed during a single week-long visit to the Houston Zoo, the number of cells available from each animal was limited, which resulted in some stimulation conditions (e.g., ionomycin) being tested in fewer elephants than others. For the same logistical reasons, stimulant concentrations for Asian elephant experiments could not be optimized through pilot titrations. Therefore, concentrations were selected within the effective range previously established for African elephant heterophils. As the experiments for the two species were not performed side-by-side, these datasets were not intended for direct species comparisons.

For real-time microscopy, MPO-FITC and SYTOX AADvanced were added to RPMI media with 0.05% heat inactivated FBS + 0.01 M HEPES. PMA, LPS, and Ionomycin were added at the concentrations shown in the respective figures in a 96-well plate with media containing dye (MPO-FITC and SYTOX AADvanced). SYTOX is a DNA stain that labels extracellular DNA and DNA from cells with compromised cell membranes. African elephant (*n* = 2) heterophils were then stained for viability and counted by Trypan Blue exclusion. Cells were seeded at 10,000 cells/well in 96-well plates. In each real-time microscopy assay, heterophils from an individual elephant were tested in quadruplicate and heterophils from each of the two African elephants available for testing were tested twice from temporally separated blood collections. The plates were then imaged in the Incucyte (Sartorius) every 15 min, capturing 2 images per well, at 650 ms GFP and 500 ms RFP. Data were analyzed by measuring area of overlap for MPO-FITC and SYTOX by phase confluence using Incucyte 2023 Rev2 software. Representative results are displayed in Fig. 2D.

### Tissue samples

Tissue samples from Asian elephants with lethal EEHV-HD and from Asian elephants that died of other causes were collected at necropsy from elephants housed in zoological institutions in the United States (Table 1). All animals were under professional veterinary care, and tissues were obtained opportunistically following death. Sample collection and use were conducted according to participating zoos’ Animal Welfare and Research Committees and Association of Zoos and Aquariums (AZA) Elephant Research and Necropsy Protocol with endorsement from the AZA Elephant Taxon Advisory Group and Species Survival Plan (TAG/SSP) Steering Committee. Samples were provided by Rosamond Gifford Zoo, Columbus Zoo, San Antonio Zoo, ABQ BioPark Zoo, Denver Zoo, Santa Barbara Zoo, Oklahoma City Zoo, Dickerson Park Zoo, and others. We have complied with all relevant ethical regulations for animal use. Tissues were frozen immediately or fixed in formalin, sectioned to 5 μm thickness, and mounted on slides.

### Immunofluorescence

Tissue samples were stained with rabbit anti-human citrullinated histone H3 (1:400, ab5103; Abcam) and goat anti-human/mouse MPO (1:67, AF3667; R&D Systems) with AlexaFluor488-conjugated anti-rabbit and DyLight 649-conjugated anti-goat secondary antibodies. ProLong Glass Antifade Mountant formulated with NucBlu (Hoechst 33342) was used to stain DNA (P36981; Invitrogen). Images were captured by confocal microscopy (Leica SP8) and analyzed using FIJI/Image-J (version 2.1.0/1.53c; NIH). Myeloperoxidase (MPO) was detected using a polyclonal anti-human MPO antibody validated to recognize MPO across multiple mammalian species, including human and mouse. Although elephant MPO is not fully identical to human MPO (Supplementary Fig. 2), the polyclonal nature of the antibody and conservation of key epitopes support cross-reactivity and the staining patterns for elephant heterophils were consistent with those observed for human and mouse samples; the antibody was used at a dilution within the manufacturer’s recommended range for immunocytochemistry relative to stock concentration. Additionally, MPO signal was localized around the nucleus, consistent with granule localization in resting human neutrophils^84,85^. To assess the suitability of the citrullinated histone H3 (citH3) antibody for use in elephant samples, we performed a sequence alignment of human, African elephant, and Asian elephant histone H3. The N-terminal tail of H3 encompassing the citrullination sites targeted by citH3 antibodies (Arg2, Arg8, Arg17, and Arg26) was fully conserved between species (Supplementary Fig. 3), supporting cross-reactive recognition of citrullinated H3 in elephant cells.

### Imaging and quantification

All samples were deidentified prior to imaging to blind the results during image collection and quantification. First, whole slide immunofluorescence images were collected by confocal microscopy (Leica SP8) by one individual. A second individual then reviewed the slides and selected ten 4-by-4 tiled representative sections with the most citrullinated histone H3 and MPO staining. A third individual then collected high-resolution images of each 4-by-4 section by confocal microscopy. Each blinded section was analyzed using FIJI/Image-J’s cell counter plug-in (version 2.1.0/1.53c; NIH) to calculate the percent of citrullinated histone H3-positive cells, relative to Hoechst.

### Statistics and reproducibility

Bars and error bars in all graphs represent means +/− standard error of the mean (SEM). For experiments in which heterophils from the same elephant were tested under more than two matched conditions, data were analyzed using repeated-measures one-way ANOVA. Datasets were presented descriptively without statistical analysis when two or fewer individuals were tested. For experiments designed to test specific a priori hypotheses regarding inhibitor efficacy, planned paired comparisons were performed using paired two-tailed t-tests with Holm–Šídák correction. Real-time fluorescence was quantified as area under the curve (AUC) per well (*n* = 4 technical replicates per condition). AUC values were compared by one-way ANOVA with Dunnett’s multiple comparisons test versus 0 µg/mL. For tissue analyses (Fig. 5), comparisons between EEHV-HD-negative and EEHV-HD-positive samples were performed using unpaired two-tailed t-tests with Welch’s correction. *p* < 0.05 was considered statistically significant.

Experiments were performed using primary elephant heterophils obtained from multiple individual animals where sample availability permitted. Biological replicates are defined as independent samples collected from different elephants or from the same elephant on separate days, and technical replicates represent parallel wells within a single experiment. Key findings were reproduced across independent animals and experimental replicates where feasible. Due to the limited availability of samples, some experiments were performed with small numbers of animals (*n* ≤ 2) and are presented descriptively without statistical analysis; these limitations are explicitly indicated in the figure legends. No data were excluded, and all experiments meeting quality and viability criteria were included in the analysis.

### Reporting summary

Further information on research design is available in the Nature Portfolio Reporting Summary linked to this article.

## Supplementary information

Supplementary Information

Description of Additional Supplementary Materials

Supplementary Data

Reporting Summary

## Data Availability

All data generated or analyzed during this study are included in this published article and its Supplementary Information files. Source data underlying the figures are provided in the Supplementary data file.
