# Supplementary material for: Neutrophil extracellular traps offer a new therapeutic target for elephant endotheliotropic herpes virus-hemorrhagic disease (EEHV-HD)
Source: Commun Biol. 2026 May 30;9:1147. doi: 10.1038/s42003-026-10375-w (PMC13518930; doi:10.1038/s42003-026-10375-w)
Supplement: Supplementary file 1 [file 42003_2026_10375_MOESM1_ESM.pdf]

## Supplemental Figure 1

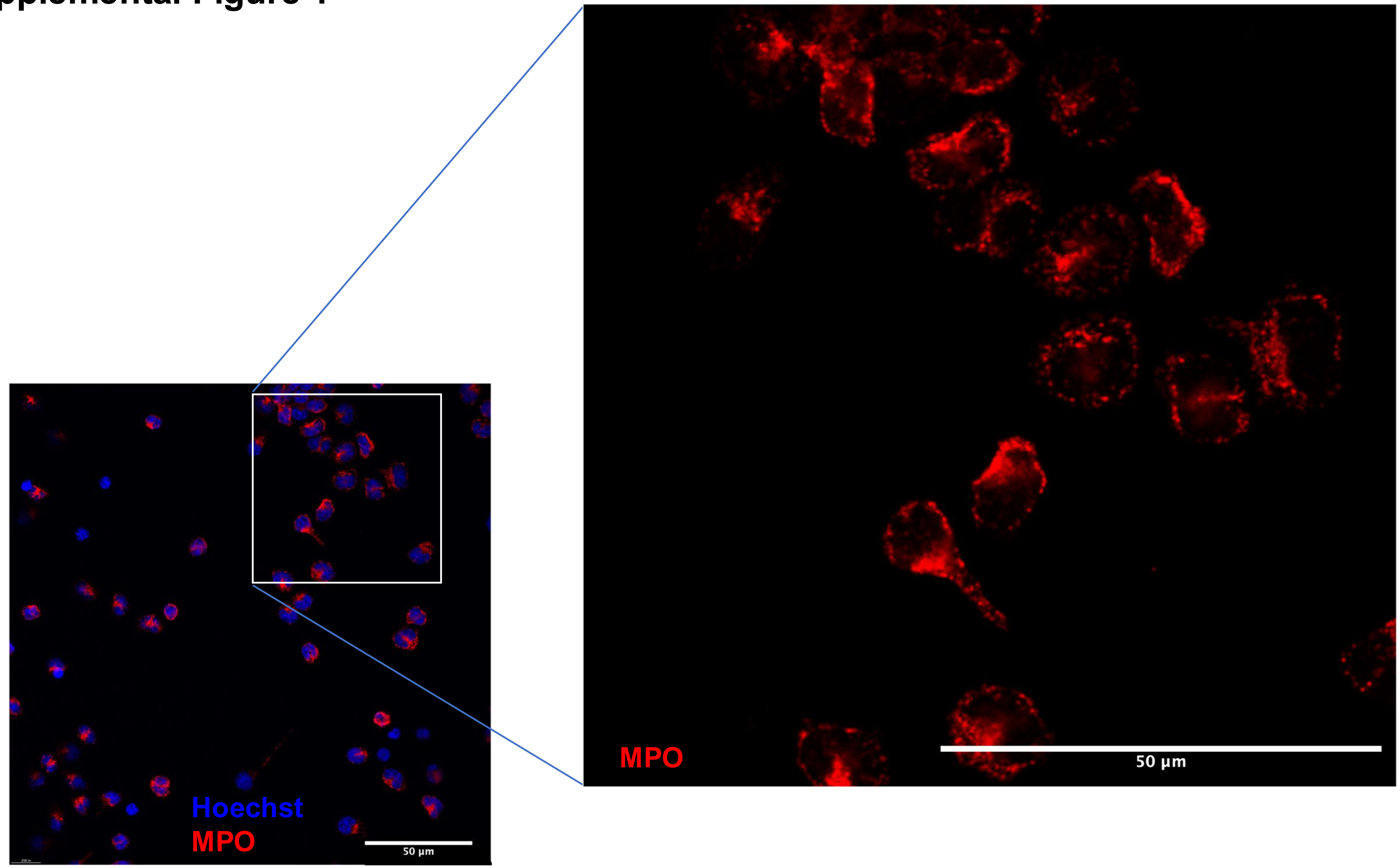


**Supplementary Figure 1**: Granular MPO staining in elephant heterophils. Representative immunofluorescence images of elephant heterophils stained for myeloperoxidase (MPO, red) and DNA (Hoechst, blue). The left panel shows a lower-magnification field of view, with a boxed region indicating the area shown at higher magnification in the right panel. High-resolution imaging reveals punctate, granular MPO staining localized to the cytoplasm surrounding the nucleus, consistent with the known granular distribution of MPO in neutrophils from other mammalian species, supporting the suitability of the antibody for detecting elephant MPO.

## Supplemental Figure 2

```
Human MPO: 1              mgvpffsslrcmvdlgpcwaggltaeMKLLLALAGLLAILATPQPSEGAAPAVLGEVDTSLVLSSMEEAKQLVDKAYKER  80
African Elephant MPO: 1   --------------------------MKLLLALAGLLATLAMPPPSEGTTPAVLREVETSVVLTSMEEAKRLVDKAYKER  54
Asian Elephant MPO: 1     --------------------------MKLLLALAGLLATLAMPPPSEGTTPAVLREVETSVVLTSMEEAKRLVDKAYKER  54

Human MPO: 81             RESIKQRLRSGSASPMELLSYFKQPVAATRTAVRAADYLHVALDLLERKLRSLWRRPFNVTDVLTPAQLNVLSKSSGCAY  160
African Elephant MPO: 55  RESIKQRLRSRLASPMELLSYFKQPVAATRTAVRAADYLHVALSLLEGKLRSLWPRPFNVTDVLTPSQLNLLWKSSGCAY  134
Asian Elephant MPO: 55    RESIKQRLRSRLASPMELLSYFKQPVAATRTAVRAADYLHVALSLLEGKLRSLWPRPFNVTDVLTPSQLNLLWKSSGCAY  134

Human MPO: 161             QDVGVTCPEQDKYRTITGMCNNRRSPTLGASNRAFVRWLPAEYEDGFSLPYGWTPGVKRNGFPVALARAVSNEIVRFPTD  240
African Elephant MPO: 135  QDVAVTCPERDKYRTITGKCNNRRSPTLGSSNRPFVRWLPAEYEDGFSLPFGWTPGVKRGGFPVPLTRAVSNAIVRFPTE  214
Asian Elephant MPO: 135    QDVAVTCPERDKYRTITGKCNNRRSPTLGSSNRPFVRWLPAEYEDGFSLPFGWTPGVKRGGFPVPLTRAVSNAIVRFPTE  214

Human MPO: 241             QLTPDQERSLMFMQWGQLLDHDLDFTPEPAARASFVTGVNCETSCVQQPPCFPLKIPPNDPRIKNQADCIPFFRSCPACP  320
African Elephant MPO: 215  QLTPDRERALMFMQWGQLLDHDLDFTPEPAARASFTTGLNCETSCLQQPPCFPLKIPPNDPRIKNQRDCIPFFRSCPACT  294
Asian Elephant MPO: 215    QLTPDRERALMFMQWGQLLDHDLDFTPEPAARASFTTGLNCETSCLQQPPCFPLKIPPNDPRIKNQRDCIPFFRSCPACT  294

Human MPO: 321             GSNITIRNQINALTSFVDASMVYGSEEPLARNLRNMSNQLGLLAVNQRFQDNGRALLPFDNLHDDPCLLTNRSARIPCFL  400
African Elephant MPO: 295  GGNITIRNQINALTSFVDASMVYGSEDPLARDLRNLTNQLGLLRVNDRFRDNGRALLPFDKLHDDPCLLTNRSVRIPCFL  374
Asian Elephant MPO: 295    GGNITIRNQINALTSFVDASMVYGSEDPLARDLRNLTNQLGLLRVNDRFRDNGRALLPFDKLHDDPCLLTNRSVRIPCFL  374

Human MPO: 401             AGDTRSSEMPELTSMHTLLLREHNRLATELKSLNPRWDGERLYQEARKIVGAMVQIITYRDYLPLVLGPTAMRKYLPTYR  480
African Elephant MPO: 375  AGDTRSSEMPELTSMHTLLVREHNRLATELKRLNPRWDGERLYQEARKIVGAMVQIITYRDYLPLVLGPEAFRKYLPQYR  454
Asian Elephant MPO: 375    AGDTRSSEMPELTSMHTLLVREHNRLATELKRLNPRWDGERLYQEARKIVGAMVQIITYRDYLPLVLGPEAFRKYLPQYR  454

Human MPO: 481             SYNDSVDPRIANVFTNAFRYGHTLIQPFMFRLDNRYQPMEpnpRVPLSRVFFASWRVVLEGGIDPILRGLMATPAKLNRQ  560
African Elephant MPO: 455  GYNESVDPRIANVFTNAFRYGHTLIQPFMFRLDNRYQLIS---RVPLSEVFFATWRVVLEGGIDPILRGLMASPAKLNRQ  531
Asian Elephant MPO: 455    GYNESVDPRIANVFTNAFRYGHTLIQPFMFRLDNRYQLIS---RVPLSEVFFATWRVVLEGGIDPILRGLMASPAKLNRQ  531

Human MPO: 561             NQIAVDEIRERLFEQVMRIGLDLPALNMQRSRDHGLPGYNAWRRFCGLPQPETVGQLGTVLRNLKLARKLMEQYGTPNNI  640
African Elephant MPO: 532  NQIVVDEVRDRLFKQVMRIGLDLPALNMQRSRDHGLPGYNAWRRFCGLSQPNTVGELGTVMKNLELARKLMAQYGTPNNI  611
Asian Elephant MPO: 532    NQIVVDEVRDRLFKQVMRIGLDLPALNMQRSRDHGLPGYNAWRRFCGLSQPNTVGELGTVMKNLELARKLMAQYGTPNNI  611

Human MPO: 641             DIWMGGVSEPLKRKGRVGPLLACIIGTQFRKLRDGDRFWWENEGVFSMQQRQALAQISLPRIICDNTGITTVSKNNIFMS  720
African Elephant MPO: 612  DIWMGGVAEPLQPNGRVGPLLACIIGTQFRKLRDGDRFWWENNGVFSTQQRQALAKISLPRIICDNTGITTVSKNNIFMS  691
Asian Elephant MPO: 612    DIWMGGVAEPLQPNGRVGPLLACIIGTQFRKLRDGDRFWWENNGVFSTQQRQALAKISLPRIICDNTGITTVSKNNIFMS  691

Human MPO: 721             NSYPRDFVNCSTLPALNLASWREAS  745
African Elephant MPO: 692  HMYPRDFVSCRTLPALDLTPWRDRA  716
Asian Elephant MPO: 692    HMYPRDFVSCRTLPALDLTPWRDRA  716
```

**Supplementary Figure 2**: Amino acid sequence alignment of human myeloperoxidase (MPO) (NP_000241.1), African elephant MPO (XP_003414426.2), and Asian elephant myeloperoxidase MPO (XP_049717144.1).

## Supplemental Figure 3

| | | | |
|---|---|---|---|
| Human H3.1: | 1 | MARTKQTARKSTGGKAPRKQLATKAARKSAPATGGVKKPHRYRPGTVALREIRRYQKSTELLIRKLPFQRLVREIAQDFK | 80 |
| Asian elephant H3: | 1 | MARTKQTARKSTGGKAPRKQLATKAARKSAPATGGVKKPHRYRPGTVALREIRRYQKSTELLIRKLPFQRLVREIAQDFK | 80 |
| African elephant H3: | 1 | MARTKQTARKSTGGKAPRKQLATKAARKSAPATGGVKKPHRYRPGTVALREIRRYQKSTELLIRKLPFQRLVREIAQDFK | 80 |
| Human H3.1: | 81 | TDLRFQSSAVMALQEACEAYLVGLFEDTNLCAIHAKRVTIMPKDIQLARRIRGERA | 136 |
| Asian elephant H3: | 81 | TDLRFQSSAVMALQEASEAYLVGLFEDTNLCAIHAKRVTIMPKDIQLARRIRGERA | 136 |
| African elephant H3: | 81 | TDLRFQSSAVMALQEASEAYLVGLFEDTNLCAIHAKRVTIMPKDIQLARRIRGERA | 136 |

**Supplementary Figure 3**: Amino acid sequence alignment of human histone H3 (H3) (NP_003522.1), African elephant H3 (XP_064138579.1), and Asian elephant histone H3 (XP_049736263.1). The boxed region denotes the N-terminal tail of H3 (residues 1-40 or the mature protein) encompassing the epitope recognized by citrullinated histone H3 (citH3) antibodies. Arrows indicate the arginine residues (Arg2, Arg8, Arg17, and Arg26) that undergo citrullination.
