# Supplementary material for: Neutrophil extracellular traps offer a new therapeutic target for elephant endotheliotropic herpes virus-hemorrhagic disease (EEHV-HD)
Source: Commun Biol. 2026 May 30;9:1147. doi: 10.1038/s42003-026-10375-w (PMC13518930; doi:10.1038/s42003-026-10375-w)
Supplement: Supplementary file 2 [file 42003_2026_10375_MOESM2_ESM.pdf]

Description of Additional Supplementary Files

File name: Supplementary Data 1

Description: The source data behind the graphs in the paper.
