# Supplementary material for: Neutrophil extracellular traps offer a new therapeutic target for elephant endotheliotropic herpes virus-hemorrhagic disease (EEHV-HD)
Source: Commun Biol. 2026 May 30;9:1147. doi: 10.1038/s42003-026-10375-w (PMC13518930; doi:10.1038/s42003-026-10375-w)
Supplement: Supplementary file 3 [file 42003_2026_10375_MOESM4_ESM.pdf]

nature portfolio

Corresponding author(s): Lisa M. Abegglen

Last updated by author(s): Feb 4, 2026

Reporting Summary

Nature Portfolio wishes to improve the reproducibility of the work that we publish. This form provides structure for consistency and transparency in reporting. For further information on Nature Portfolio policies, see our Editorial Policies and the Editorial Policy Checklist.

Statistics

For all statistical analyses, confirm that the following items are present in the figure legend, table legend, main text, or Methods section.

n/a	Confirmed	
☐	☒	The exact sample size (*n*) for each experimental group/condition, given as a discrete number and unit of measurement
☐	☒	A statement on whether measurements were taken from distinct samples or whether the same sample was measured repeatedly
☐	☒	The statistical test(s) used AND whether they are one- or two-sided<br>*Only common tests should be described solely by name; describe more complex techniques in the Methods section.*
☒	☐	A description of all covariates tested
☐	☒	A description of any assumptions or corrections, such as tests of normality and adjustment for multiple comparisons
☐	☒	A full description of the statistical parameters including central tendency (e.g. means) or other basic estimates (e.g. regression coefficient) AND variation (e.g. standard deviation) or associated estimates of uncertainty (e.g. confidence intervals)
☐	☒	For null hypothesis testing, the test statistic (e.g. F, t, r) with confidence intervals, effect sizes, degrees of freedom and P value noted<br>*Give P values as exact values whenever suitable.*
☒	☐	For Bayesian analysis, information on the choice of priors and Markov chain Monte Carlo settings
☒	☐	For hierarchical and complex designs, identification of the appropriate level for tests and full reporting of outcomes
☒	☐	Estimates of effect sizes (e.g. Cohen's d, Pearson's r), indicating how they were calculated

Our web collection on statistics for biologists contains articles on many of the points above.

Software and code

Policy information about availability of computer code

Data collection	Image acquisition was performed using manufacturer-provided microscope control software (Leica) and the IncuCyte live-cell imaging system software (Sartorius) for real-time fluorescence imaging, according to standard operating procedures. No custom code was used for data collection.
Data analysis	Data analysis and statistical testing were performed using GraphPad Prism (GraphPad Software). Image processing and quantification were conducted using standard analysis tools provided with the imaging software and ImageJ/Fiji. No custom code or custom algorithms were used for data analysis.

For manuscripts utilizing custom algorithms or software that are central to the research but not yet described in published literature, software must be made available to editors and reviewers. We strongly encourage code deposition in a community repository (e.g. GitHub). See the Nature Portfolio guidelines for submitting code & software for further information.

Data

Policy information about availability of data

All manuscripts must include a data availability statement. This statement should provide the following information, where applicable:

- Accession codes, unique identifiers, or web links for publicly available datasets
- A description of any restrictions on data availability
- For clinical datasets or third party data, please ensure that the statement adheres to our policy

Data is provided in a supplementary file.

Research involving human participants, their data, or biological material

Policy information about studies with human participants or human data. See also policy information about sex, gender (identity/presentation), and sexual orientation and race, ethnicity and racism.

Reporting on sex and gender	This study did not involve human participants, human data, or human biological material. For elephant samples, sex was recorded and reported where available for tissue specimens (Table 1). For in vitro heterophil experiments, the number of elephants tested was limited by sample availability, and experiments were not powered to assess sex-dependent differences in NET formation. As such, sex was not considered as a biological variable in statistical analyses of in vitro experiments.
Reporting on race, ethnicity, or other socially relevant groupings	This study did not involve human participants, human data, or human biological material. Accordingly, race, ethnicity, or other socially constructed groupings were not applicable and were not collected or analyzed.
Population characteristics	This study did not involve human research participants. Relevant biological characteristics of elephant samples, including species, age, sex (where available), disease status, and tissue source, are reported in Table 1 and the Methods.
Recruitment	This study did not involve recruitment of human participants. Elephant samples were obtained opportunistically through routine veterinary care and necropsy at participating zoological institutions.
Ethics oversight	Sample collection was approved by the internal review committees of participating zoological institutions in accordance with institutional policies governing veterinary procedures and post-mortem tissue collection. No experimental interventions were performed on live animals.

Note that full information on the approval of the study protocol must also be provided in the manuscript.

Field-specific reporting

Please select the one below that is the best fit for your research. If you are not sure, read the appropriate sections before making your selection.

☒ Life sciences ☐ Behavioural & social sciences ☐ Ecological, evolutionary & environmental sciences

For a reference copy of the document with all sections, see nature.com/documents/nr-reporting-summary-flat.pdf

Life sciences study design

All studies must disclose on these points even when the disclosure is negative.

Sample size	Sample sizes were determined by the availability of elephants and tissues and the logistical constraints associated with rapid sample processing. No a priori statistical power calculations were performed. Exact sample sizes (number of elephants and technical replicates) are reported for each experiment in the Methods, Results, and figure legends.
Data exclusions	No data were excluded from analyses. All samples collected and processed within viability and quality constraints were included. Where sample sizes were too small to support formal statistical testing, data are presented descriptively and this is explicitly stated in the figure legends and Results.
Replication	Key findings were reproduced across independent elephants where sample availability permitted. In vitro experiments include technical replication within individual elephants, and biological replication across multiple elephants when feasible. Some experiments were limited to small numbers of elephants due to logistical constraints and are presented as descriptive; these limitations are clearly stated in the manuscript.
Randomization	Randomization was not performed. Samples were obtained opportunistically during routine veterinary care or necropsy, and allocation to experimental conditions was determined by sample availability and assay design rather than random assignment.
Blinding	Investigators were not blinded during sample collection. For imaging-based analyses, samples were deidentified prior to image acquisition and quantification, and image analysis was performed blinded to experimental condition.

Reporting for specific materials, systems and methods

We require information from authors about some types of materials, experimental systems and methods used in many studies. Here, indicate whether each material, system or method listed is relevant to your study. If you are not sure if a list item applies to your research, read the appropriate section before selecting a response.

Materials & experimental systems

n/a	Involved in the study
☐	☒ Antibodies
☒	☐ Eukaryotic cell lines
☒	☐ Palaeontology and archaeology
☐	☒ Animals and other organisms
☒	☐ Clinical data
☒	☐ Dual use research of concern
☒	☐ Plants

Methods

n/a	Involved in the study
☒	☐ ChIP-seq
☒	☐ Flow cytometry
☒	☐ MRI-based neuroimaging

Antibodies

Antibodies used	Primary antibodies used in this study include anti–citrullinated histone H3 (citH3) and anti–myeloperoxidase (MPO) antibodies for immunofluorescence, as detailed in the Methods section. Supplier name, catalog number, and working dilution for each antibody are provided in the Methods.
Validation	Antibodies were validated based on manufacturer-provided information and prior use in the literature. The anti-citH3 antibody targets a highly conserved N-terminal histone H3 epitope; sequence alignment between human and elephant histone H3 is provided in Supplementary Fig. 3, supporting cross-reactivity. The anti-MPO antibody is polyclonal and validated for cross-species reactivity, including in multiple mammalian species; sequence conservation between human and elephant MPO is shown in Supplementary Fig. 2. Granular perinuclear MPO staining consistent with neutrophil/heterophil granule localization is demonstrated in elephant heterophils (Supplementary Fig. 1).

Animals and other research organisms

Policy information about studies involving animals; ARRIVE guidelines recommended for reporting animal research, and Sex and Gender in Research

Laboratory animals	This study did not involve laboratory animals.
Wild animals	This study did not involve wild animals. All samples were obtained from Asian and African elephants under human care housed in zoological institutions during routine veterinary care or necropsy.
Reporting on sex	Sex was recorded for elephants where available and is reported in Table 1 for tissue samples. Due to small sample sizes and limited availability of animals, sex was not considered as a biological variable in experimental design or analysis, and sex-based comparisons were not performed for in vitro heterophil experiments.
Field-collected samples	Blood and tissue samples were obtained opportunistically during routine veterinary procedures or necropsy. No experimental manipulation of animals occurred for the purposes of this study. Samples were processed immediately or preserved according to standard protocols described in the Methods.
Ethics oversight	Sample collection and use were approved by the internal research or animal care and use oversight committees of participating institutions and were conducted in accordance with institutional and veterinary guidelines. No additional ethical approval was required, as no experimental procedures were performed on live animals specifically for this study.

Note that full information on the approval of the study protocol must also be provided in the manuscript.

Plants

Seed stocks	This study did not involve plants.
Novel plant genotypes	This study did not involve plants.
Authentication	This study did not involve plants.
